# The Physiological Effects of Deleting the Mouse *Slc30a8* Gene Encoding Zinc Transporter-8 Are Influenced by Gender and Genetic Background

**DOI:** 10.1371/journal.pone.0040972

**Published:** 2012-07-19

**Authors:** Lynley D. Pound, Suparna A. Sarkar, Alessandro Ustione, Prasanna K. Dadi, Melanie K. Shadoan, Catherine E. Lee, Jay A. Walters, Masakazu Shiota, Owen P. McGuinness, David A. Jacobson, David W. Piston, John C. Hutton, David R. Powell, Richard M. O’Brien

**Affiliations:** 1 Department of Molecular Physiology and Biophysics, Vanderbilt University Medical School, Nashville, Tennessee, United States of America; 2 Lexicon Pharmaceuticals Incorporated, The Woodlands, Texas, United States of America; 3 Barbara Davis Center for Childhood Diabetes, University of Colorado Health Sciences Center, Aurora, Colorado, United States of America; University of British Columbia, Canada

## Abstract

**Objective:**

The *SLC30A8* gene encodes the islet-specific transporter ZnT-8, which is hypothesized to provide zinc for insulin-crystal formation. A polymorphic variant in *SLC30A8* is associated with altered susceptibility to type 2 diabetes. Several groups have examined the effect of global *Slc30a8* gene deletion but the results have been highly variable, perhaps due to the mixed 129SvEv/C57BL/6J genetic background of the mice studied. We therefore sought to remove the conflicting effect of 129SvEv-specific modifier genes.

**Methods:**

The impact of *Slc30a8* deletion was examined in the context of the pure C57BL/6J genetic background.

**Results:**

Male C57BL/6J *Slc30a8* knockout (KO) mice had normal fasting insulin levels and no change in glucose-stimulated insulin secretion (GSIS) from isolated islets in marked contrast to the ∼50% and ∼35% decrease, respectively, in both parameters observed in male mixed genetic background *Slc30a8* KO mice. This observation suggests that 129SvEv-specific modifier genes modulate the impact of *Slc30a8* deletion. In contrast, female C57BL/6J *Slc30a8* KO mice had reduced (∼20%) fasting insulin levels, though this was not associated with a change in fasting blood glucose (FBG), or GSIS from isolated islets. This observation indicates that gender also modulates the impact of *Slc30a8* deletion, though the physiological explanation as to why impaired insulin secretion is not accompanied by elevated FBG is unclear. Neither male nor female C57BL/6J *Slc30a8* KO mice showed impaired glucose tolerance.

**Conclusions:**

Our data suggest that, despite a marked reduction in islet zinc content, the absence of ZnT-8 does not have a substantial impact on mouse physiology.

## Introduction

Zinc is fundamental to many biological processes and thus is tightly regulated by two types of proteins: metallothioneins, which are responsible for intracellular zinc storage and trafficking, and zinc transporters [1]. The SLC39 (Zip) family of transporters allows for cellular influx of zinc while the SLC30 (ZnT) family is responsible for efflux of zinc out of the cell or into intracellular vesicles [1]. The ten members of the ZnT family share a similar six transmembrane domain structure with a histidine rich loop located between helices IV and V, with the exception of ZnT-6, which contains a serine-rich loop, and ZnT-10, which contains a basic amino acid-rich loop [1]. *SLC30A8*, which encodes ZnT-8, is highly expressed in the pancreatic islet, in both beta [2] and alpha cells [3], and to a lesser extent in the testis, submaxillary glands, the cubical epithelium that lines the thyroid follicles and the adrenal cortex [4]. Within the beta cell ZnT-8 is thought to reside in insulin secretory granules where zinc levels are among the highest in the body [5]. There, two zinc ions complex with insulin hexamers to promote proper insulin maturation, storage and secretion [6]. Thus, it has been hypothesized that ZnT-8 is responsible for transporting zinc into the insulin secretory granules within the islet [7].

Consistent with this hypothesis, studies *in vitro* have shown that overexpression of ZnT-8 in INS-1E cells results in increased zinc content and enhanced glucose stimulated insulin secretion (GSIS) [5]. In addition, genome-wide association (GWA) studies in humans have linked a non-synonymous single nucleotide polymorphism (SNP) within the *SLC30A8* gene with increased susceptibility for the development of type 2 diabetes [8]–[11] and gestational diabetes [12]. Since beta cell failure is the key event in the transition from an insulin resistant state associated with normal glucose tolerance to type 2 diabetes [13] these observations are consistent with an important role for ZnT-8 in beta cell function. This same *SLC30A8* SNP is also associated with impaired proinsulin to insulin conversion [14] and reduced first phase insulin secretion [15], consistent with the proposed key role of ZnT-8 in insulin secretion rather than other aspects of beta cell function. Interestingly, ZnT-8 is also an autoantigen in type 1 diabetes and this same polymorphism, which results in an arginine to tryptophan mutation at amino acid residue 325, has also been shown to affect autoantibody epitope specificity [16].

Given the importance of understanding the molecular basis for the connection between ZnT-8 and altered diabetes susceptibility we recently investigated the effect of a global deletion of *Slc30a8*, in the context of mice with a mixed C57BL/6J×129SvEv genetic background, to address the hypothesis that the absence of ZnT-8 results in impaired islet function and thereby contributes to type 2 diabetes. We demonstrated that deletion of *Slc30a8* in these mixed genetic background mice resulted in a marked reduction in islet zinc content, reduced fasting plasma insulin levels and impaired GSIS from isolated *Slc30a8* knockout (KO) mouse islets [17]. Surprisingly, however, the mice displayed no defects in fasting blood glucose levels nor did they exhibit impaired glucose tolerance [17]. Three other studies have also investigated the effect of deleting the *Slc30a8* gene in mixed genetic background mice. These studies also demonstrated a significant loss of zinc within the islet [18]–[20], supporting the proposed role of ZnT-8. In contrast, however, the reported effect of *Slc30a8* deletion on insulin secretion from isolated islets varied between these studies. Wijesekara *et al.* demonstrated that GSIS was reduced in islets isolated from beta cell-specific *Slc30a8* KO mice, consistent with the results of our study, though the authors also reported no change in insulin secretion in beta cell-specific *Slc30a8* KO mice *in vivo* despite mildly impaired glucose tolerance [18]. In contrast, Lemaire *et al.* [19] found no impairment in GSIS, while Nicolson *et al.* reported an increase in GSIS from isolated *Slc30a8* KO mouse islets, though this result varied with age and gender [20]. Surprisingly, however, this same study demonstrated a reduction in insulin secretion in *Slc30a8* KO mice during an intraperitoneal glucose tolerance test (IPGTT) as well as impaired glucose tolerance [20]. The differences between these studies have been attributed to variations in the age and genetic background of the mice examined [21]. Indeed glucose metabolism and islet function differ markedly between different inbred mouse strains [22]. However, there were also marked differences in the numbers of mice analyzed in these different studies, which could be significant given the mild phenotypes reported [23]. In addition, there can also be significant variations in glucose metabolism between genetically identical lines housed within different mouse facilities such that it is possible that some of the discrepancies in the phenotype of *Slc30a8* KO mice reported by different groups are due to environmental differences [24].

Due to the variation in reported phenotypes, we sought to remove the conflicting effect of 129SvEv-specific modifier genes by examining the impact of *Slc30a8* deletion in the context of the pure C57BL/6J genetic background. Because binding of zinc to insulin is thought to be important for proper insulin maturation and crystallization and, consequently, secretion, we predicted that a lack of granular zinc would again result in impaired secretion in C57BL/6J *Slc30a8* KO mice, as we saw in our characterization of *Slc30a8* KO mice on a mixed genetic background [17]. Surprisingly, we find that, in contrast to the observations made with *Slc30a8* KO mice on a mixed genetic background, on the C57BL/6J background only female *Slc30a8* KO mice display a reduction in fasting plasma insulin. Furthermore, despite this reduction in fasting plasma insulin there is no change in fasting blood glucose, glucose tolerance or GSIS in isolated islets. Our data therefore suggest that, despite the marked loss of zinc in islet insulin secretory granules, the absence of ZnT-8 does not have a substantial impact on mouse physiology.

## Materials and Methods

### Animal Care

The animal housing and surgical facilities used for this study meet the standards set by the American Association for the Accreditation of Laboratory Animal Care standards. The Vanderbilt University Medical Center Animal Care and Use Committee approved all protocols used. Mice were maintained on either a standard rodent chow diet (LabDiet 5001; 23% protein and 4.5% fat; PMI Nutrition International) or a high fat diet (20.5% protein, 36.0% fat, 35.7% carbohydrate by mass; 60% calories from fat; Mouse Diet F3282; BioServ) as indicated. Food and water were provided *ad libitum*.

### Generation of *Slc30a8* KO Mice on a Pure C57BL/6J Genetic Background

For details on the generation of *Slc30a8* KO mice on a mixed 129/SvEv^Brd^ × C57BL/6 background see Ref. [17]. The C57BL/6J *Slc30a8* ^−/−^ congenic strain was developed utilizing a speed congenic (marker-assisted) breeding strategy [25], [26] as follows: A male *Slc30a8^−^* ^/+^ mouse on the mixed 129/SvEv^Brd^×C57BL/6 background was bred with three female C57BL/6J mice and the male offspring carrying the mutated *Slc30a8* allele and with the highest content of C57BL/6J genome, based on the analysis of microsatellite DNA, was selected for the next round of breeding with female C57BL/6J mice. Additional rounds of backcrossing were performed in the same manner. Using this approach backcrossing was complete after 6 generations, with the exception of the Y chromosome. To fix the Y chromosome an additional round of breeding was performed in which the male mouse with 100% C57BL/6J genome, based on the marker analysis, was bred with female C57BL/6J mice. Female offspring from this breeding were mated with male C57BL/6J mice such that all subsequent offspring carried the C57BL/6J Y chromosome.

Details of the speed congenic breeding strategy are as follows. A panel of 61 microsatellite markers, equally spaced throughout the genome (∼30 cM intervals), were used to differentiate the genetic background of the originating/donor (C57BL/6 and 129/SvEv) and target/recipient (NOD/ShiLtJ) mouse strains. Informative markers that distinguish between these strains were selected with the aid of a ‘panel generator’ from http://www.cidr.jhmi.edu/mouse/mmset.html. First, genomic DNA was isolated by standard proteinase K digestion protocols. Genomic DNA was then mixed with True Allele PCR Premix (Applied Biosystems, Foster City, CA) and dispensed into a panel of Mouse Mapping Primers (Applied Biosystems). The setup for the multiplexing reaction and amplification parameters were followed as per manufacturer specifications. Following PCR 4 µl of multiplexed product, 0.6 µl of GS500-ROX size standard (Applied Biosystems) and 6 µl of Hi-Di formamide (Applied Biosystems) were mixed, denatured at 94°C for 3 min and loaded onto an ABI 3100 Avant Genetic Analyzer. Chromatogram data was analyzed using GeneMapper 3.5 software (Applied Biosystems).

Once mice possessed only C57BL/6J markers, heterozygous mice were bred to generate wild type, heterozygous and knockout mice. Mice were genotyped as previously described [17].

### Immunohistochemical Staining

Pancreas tissue was fixed for 1 hr in 4% paraformaldehyde in PBS and embedded for paraffin sectioning (8 mm). Primary antisera to insulin (Guinea pig 1∶100, Dako, Denmark) and glucagon (mouse 1∶100, Sigma, St Louis, MO) were combined with a mouse monoclonal antibody raised to a 102 amino acid COOH terminal human ZnT-8 peptide (amino acids 267–369) (1∶250) [27] and detected with species specific secondary antibodies conjugated to Cy2, Cy3, Cy5 and AMCA (Jackson Laboratories). Images were acquired using an Olympus BX51 and a Pixera Pro 150 ES camera. The images were later pseudo colored for illustration: red (ZnT-8), blue (glucagon) and green (insulin), using Image-Pro Plus software (Media Cybernetics, Inc., Bethesda, MD).

### Measurement of Islet Zinc Content

The content of loosely bound zinc in isolated islets was measured as described previously [17]. Briefly, freshly isolated islets from wild type and *Slc30a8* knockout mice were washed in Ca^2+^ free Hanks Balanced Salt Solution and frozen down at −80°C in 20 islet aliquots. Islet pellets were lysed by re-suspension in 1 ml lysis buffer (1% TritonX-100 in 10 mM Tris HCl, pH 7.4). Zn^2+^ concentration in the lysate was measured using the Zn^2+^ sensitive fluorescent dye FluoZin-3 (Invitrogen). In the presence of 1.181 µM FluoZin-3 the fluorescent signal at the emission peak (516 nm) was measured in the total sample lysate using a Spectramax M5 plate reader (Molecular Devices). The fluorescent signal was compared with a standard curve generated from serial dilutions of ZnSO_4_ in lysis buffer to obtain the lysate Zn^2+^ concentration and thus Zn^2+^ content per islet. As a normalization factor, the protein content per islet was measured in the total sample lysate using the BCA protein assay (Pierce). To minimize contaminating Zn^2+^, all solutions were made in double distilled H_2_O (18.2 MΩ), avoiding the use of any glassware. Blank samples were also prepared during the islet isolation to quantify any additional Zn^2+^ contamination.

### Phenotypic Analysis of Fasted Mice

Phenotypic analysis was performed on 6 h fasted mice at ∼16 weeks of age as described previously [17]. Briefly, mice were weighed after 5 h fasting and then allowed to recover for one hour prior to being anaesthetized using isoflurane and bled from the retro-orbital venous plexus. Whole blood glucose concentrations were determined using an Accu-Check Advantage monitor (Roche). EDTA was added to blood samples prior to isolation of plasma by centrifugation and trasylol (aprotinin; 5 µl; Bayer Health Care) was then added to plasma to prevent proteolysis of glucagon. Cholesterol was assayed using the cholesterol reagent kit (Raichem), whereas triacylglycerol and glycerol were assayed using a serum triacylglycerol determination kit (Sigma). Insulin and glucagon levels were quantified using radioimmunoassay (Millipore) by the Vanderbilt Diabetes Research and Training Center Hormone Assay Core. Proinsulin was measured using the Rat/Mouse Proinsulin ELISA kit (Mercodia) according to manufacturer’s instructions.

### Intraperitoneal and Oral Glucose Tolerance Tests

Intraperitoneal glucose tolerance tests (IPGTTs) and oral glucose tolerance tests (OGTTs) on 6 hr fasted conscious mice at ∼4, ∼20 and ∼22 weeks of age, respectively, were performed as previously described [17]. Mice were fasted for 5 hours, weighed and then rested for 1 hour. Mice were then anesthetized using isoflurane and their tails were clipped. After ∼15 mins for recovery from anesthesia blood samples were isolated from the tail vein to obtain basal glucose levels. Hyperglycemia was then induced either by injection or gavage with 2.0 g/kg body weight dextrose in sterile PBS and blood glucose was measured in tail vein samples using a glucose meter (Freestyle; Abbott Diabetes Care Inc., Alameda, CA, USA).

### Islet Isolation, GSIS and Arginine-stimulated Glucagon Secretion Assays

Islets from ∼18-week-old mice were isolated [28] and cultured [17] as described previously. Briefly, isolated islets were rinsed and then incubated in RPMI-1640 medium containing 11 mM glucose overnight at 37°C. The next day islets were transferred into medium with 5 mM glucose and allowed to equilibrate for 1 h at 37°C. Following this equilibration period, for the insulin secretion assays, 20–30 islet equivalents (IEQs) were incubated in 5 ml of medium with 5 or 11 mM glucose for 30 min at 37°C. Following the same equilibration period, for the glucagon secretion assays, islets were incubated in medium with 5 mM glucose or 2 mM glucose with 20 mM arginine for 30 min at 37°C. At the end of the static incubations, islets were collected, washed and extracted in 0.2 ml of acid alcohol for 48 h at 4°C. The medium from the static incubations was centrifuged at 600 g for 1 min at 4°C. Islet extracts and static incubation media supernatants were stored at −80°C until assayed for insulin or glucagon as described above.

### Insulin Tolerance Tests

Insulin tolerance tests were performed on ∼18 week old mice as described [29], [30]. Briefly, male and female mice were weighed following a 4 hour fast and then allowed to recover for 1 hour. Mice were then anesthetized using isoflurane and their tails were clipped. After ∼15 mins for recovery from anesthesia blood samples were isolated from the tail vein to obtain basal glucose levels. Mice were then injected with a 0.75 U/kg body weight dose of insulin and blood glucose was then measured in tail vein samples using a glucose meter (Freestyle; Abbott Diabetes Care Inc., Alameda, CA, USA) over a 60 minute period.

### Analysis of *SLC30* Gene Expression by Quantitative RT-PCR in 9–23 Week Old Human Fetal Pancreas and Adult Human Islets

*SLC30* gene expression was analyzed using quantitative Real time PCR (Q-RT-PCR). Briefly, RNA was isolated from 9–23 week old fetal pancreas and adult human islets as previously described [31]. cDNA was then prepared from total RNA (1 µg) using the iScript cDNA synthesis kit (Bio-Rad Laboratories, Hercules, CA). cDNA samples equivalent to 0.1 µg of the original RNA sample were used as templates for amplification in a 5′ nuclease assay based system using FAM® dye labeled Taqman MGB probes for selected *SLC30* genes (Applied Biosystems, Foster City, CA, USA) and a 96-well ABI 7000 PCR system instrument. The glyceraldehyde-3-phosphate dehydrogenase (*GAPDH*) gene was selected for sample normalization based on preliminary experiments with the ABI control plate (part number 430 9199). The Cycle Threshold values (CT) were measured in triplicate and the samples were re-normalized to the CT values from 9 week old pancreas samples (control group/calibrator) and data was quantified using the 2– Delta Delta CT method (ABI User Bulletin #2).

### Electron Microscopy

Primary fixation was performed by pancreas perfusion [32] with Karnovsky’s fixative (2.5% glutaraldehyde and 2% paraformaldehyde in 0.1 M sodium cacodylate buffer with 1% calcium chloride, pH 7.4). Post fixation was performed with 1% osmium tetroxide, followed by dehydration with a graded series of ethanols, and embedding of tissue with Epon resin. After islets were located on 500 nm sections, 60–70 nm sections were collected on grids, stained with uranyl acetate and lead citrate, and examined by transmission electron microscopy with a Philips CM-12 typically operated at 80 kV. Images were obtained with a 2 k×2 k CCD AMT 542 digital camera. The data were analyzed in a blinded fashion by an expert in EM who did not know the genotype of the animals from which pancreas was isolated.

### Statistical Analyses

Data were analyzed using a two sample Student’s *t* test and equal variance was assumed. The level of significance was as indicated using two-sided tests except for *in vitro* glucose-stimulated insulin secretion experiments where a one-sided test was used.

## Results

### Generation of C57BL/6J *Slc30a8* KO Mice

*Slc30a8* KO mice were initially generated on a mixed 129SvEv^Brd^ × C57BL/6J genetic background [17]. Using a speed congenic breeding strategy, mice were backcrossed onto a pure C57BL/6J genetic background.

Genotype analysis of 360 three week old pups generated by cross breeding C57BL/6J *Slc30a8^−^* ^/+^ mice demonstrated that 88 mice were *Slc30a8* ^+/+^, 184 were *Slc30a8^−^* ^/+^, and 88 were *Slc30a8^−^* ^/−^, a distribution close to the expected pattern for Mendelian inheritance. The ratio of male to female mice was 201∶159. Cross breeding experiments revealed that both male and female *Slc30a8* ^−/−^ mice are fertile. Furthermore, as with mice on a mixed genetic background, no gross anatomical or behavioral abnormalities were observed in *Slc30a8* KO mice compared to wild type (WT) or heterozygous mice on the C57BL/6J genetic background.

**Figure 1:**
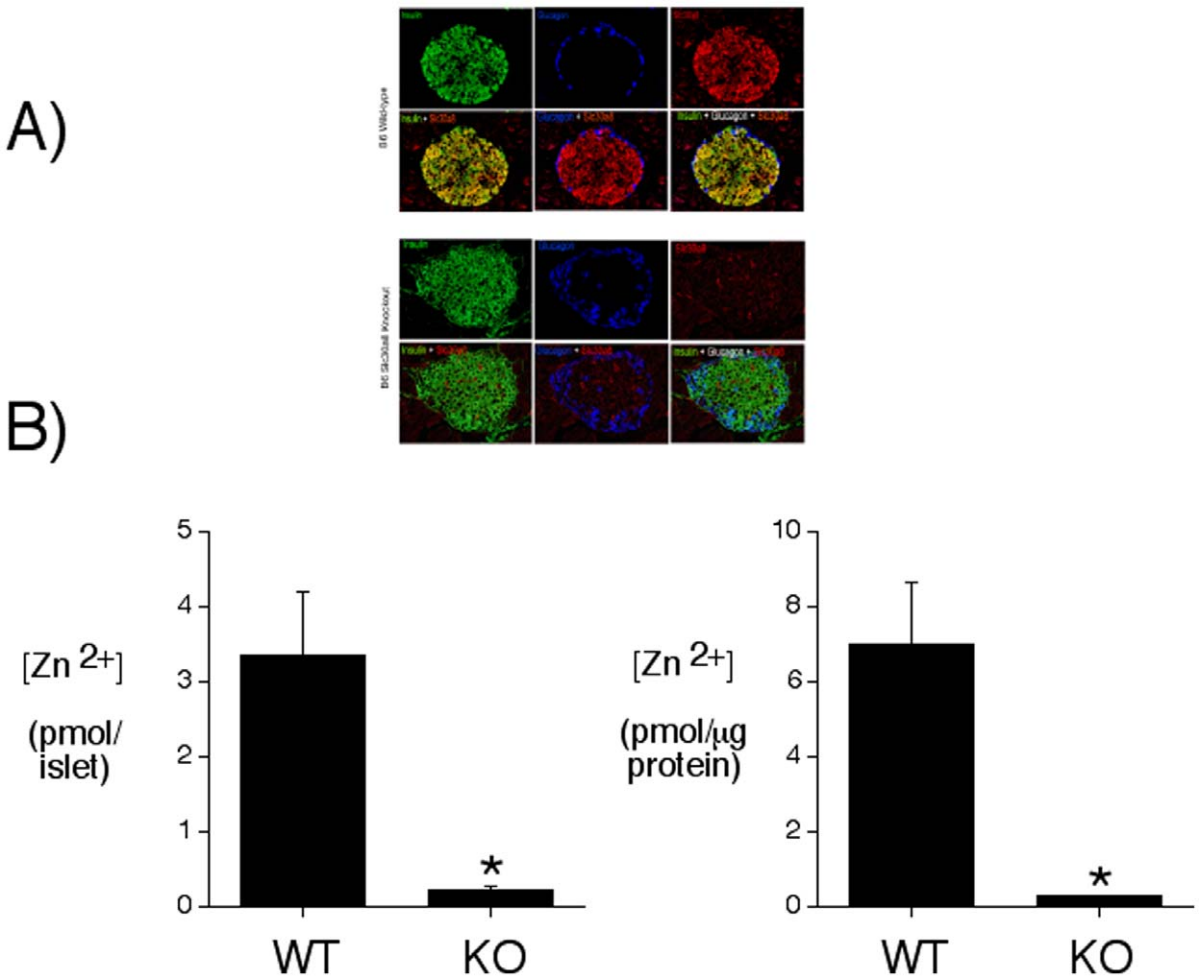
Biochemical characterization of C57BL/6J *Slc30a8* KO mice. **Panel A:** Immunohistochemical staining of wild type and *Slc30a8* KO mouse pancreas with antisera raised to insulin, glucagon, and ZnT-8 was performed as described in Materials and Methods. Representative pictures are shown. WT, wild type; KO, knockout. **Panel B:** Zinc content in isolated islets was determined as described in Material and Methods. Results represent the mean ± S.E.M. (n = 3 independent islet preparations for each genotype with each islet preparation assayed in quintuplicate). *p<0.05 *versus* WT.

To confirm that the targeting strategy had abolished ZnT-8 expression immunohistochemical staining was performed on pancreas sections prepared from a *Slc30a8* KO mouse and a wild type littermate. Figure 1A shows that ZnT-8 was detected in both alpha and beta cells in wild type but not ZnT-8 knockout mouse islets. The absence of ZnT-8 is consistent with analyses of zinc content in isolated islets using an assay that detects free or loosely bound zinc (Fig. 1B). Figure 1B shows that zinc content was markedly reduced in islets isolated from *Slc30a8* knockout mice relative to those isolated from wild type mice. The concentration of zinc detected in wild type islets was similar to that previously reported in the islet-derived INS1 cell line [5] and in islets isolated from *Slc30a8* KO mice on a mixed genetic background [17].

### Phenotypic Characterization of Male C57BL/6J *Slc30a8* KO Mice Following a Glucose Challenge

On a mixed genetic background the effect of deleting the *Slc30a8* gene was more apparent in male mice [17], thus we initially analyzed the phenotype of male *Slc30a8* KO mice on the pure C57BL/6J genetic background.

**Figure 2:**
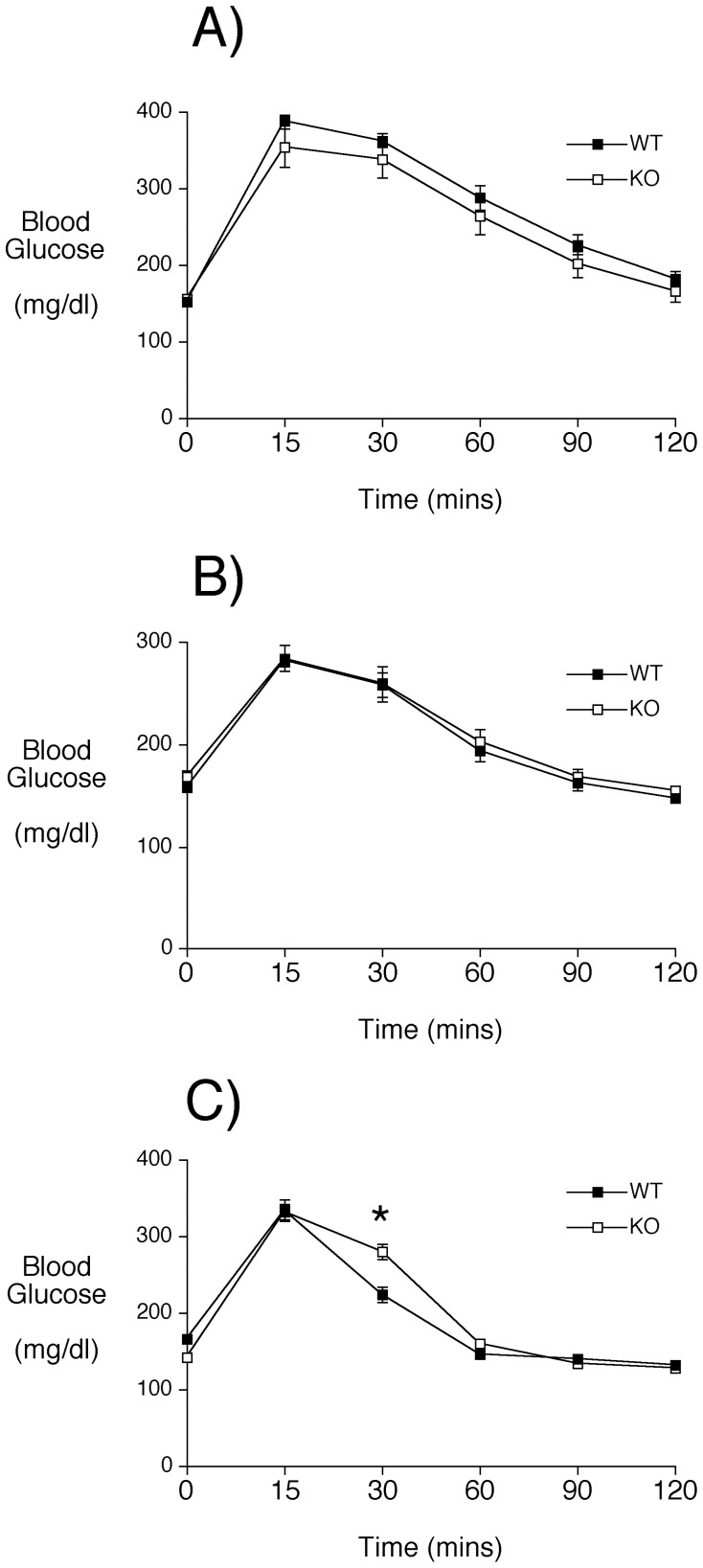
Analysis of glucose tolerance in male C57BL/6J *Slc30a8* KO mice *in vivo*. Intraperitoneal (Panels **A** and **C**) and oral (Panel **B**) glucose tolerance tests were performed on 6 hr fasted conscious C57BL/6J WT (closed symbols) and *Slc30a8* KO (open symbols) male mice as described in Materials and Methods. The IPGTT results in Panel **A** show the mean glucose concentrations ± S.E.M. in WT (n = 11; mean age ∼20 weeks) and *Slc30a8* KO (n = 9; mean age ∼20 weeks) animals. The OGTT results in Panel **B** show the mean glucose concentrations ± S.E.M. in WT (n = 16; mean age ∼22 weeks) and *Slc30a8* KO (n = 9; mean age ∼22 weeks) animals. The IPGTT results in Panel **C** show the mean glucose concentrations ± S.E.M. in WT (n = 17; mean age ∼4 weeks) and *Slc30a8* KO (n = 19; mean age ∼4 weeks) animals. *p<0.05 *versus* WT.

We first performed IPGTTs to gain insight into islet function *in vivo*. Following a 6 hour fast, male mice were injected with glucose (2 mg/g body weight) and glycemia was measured over a 120 min period. As in male *Slc30a8* KO mice on a mixed genetic background [17], no defect in glucose tolerance was observed (Fig. 2A). Furthermore, male *Slc30a8* KO mice also displayed normal glucose tolerance when glucose was administered by gavage (Fig. 2B). Thus, the results of glucose tolerance tests are consistent with the phenotype observed on a mixed genetic background [17] and, most significantly, the conclusion that global deletion of the *Slc30a8* gene has little effect on whole body glucose metabolism. In contrast with these results obtained in older (∼20–22 weeks old) male mice, a small impairment in glucose tolerance was observed when IPGTTs were performed on younger (∼4 weeks old) male mice (Fig. 2C). This suggests that a mild phenotype exists in younger animals that recedes with age, similar to the observations of Nicolson et al. [20] in animals with a global *Slc30a8* deletion analyzed on a mixed genetic background.

### Phenotypic Characterization of Fasted Male C57BL/6J *Slc30a8* KO Mice

We next investigated the phenotype of male C57BL/6J *Slc30a8* KO mice following a 6 hour fast. As with male *Slc30a8* KO mice on a mixed genetic background [17], no marked differences in body weight or length, fasting blood glucose or plasma, cholesterol, glycerol, triglycerides or glucagon were observed (Table 1). But surprisingly, male C57BL/6J *Slc30a8* KO mice also displayed normal fasting plasma insulin levels (Table 1) in marked contrast to the ∼50% reduction observed with male *Slc30a8* KO on the mixed genetic background [17]. This difference between our present studies and our previous studies [17] is unlikely to be due to environmental differences given that the animals were housed in the same facility. Instead this result suggests that the phenotype of male *Slc30a8* KO mice is dependent upon 129SvEv-specific modifier genes.

**Table 1:** Phenotypic characterization of fasted C57BL/6J *Slc30a8* KO mice.

Gender & Genotype	Weight(g)	Length(mm)	Glucose(mg/dl)	Cholesterol(mg/dl)	Triglyceride(mg/dl)	Glycerol(mg/dl)	Insulin(ng/ml)	Proinsulin(pg/ml)	Glucagon(pg/ml)
Female WT	20.1±0.3 (15)	97.4±0.3 (14)	113.7±4.0 (15)	63.1±2.3 (13)	46.6±2.0 (12)	2.9±0.2 (12)	0.57±0.04 (15)	25.4±3.8 (17)	48.4±6.7 (10)
Female^−/+^	20.0±0.2 (33)	97.1±0.3 (33)	113.5±2.1 (31)	58.8±1.7 (20)	44.2±1.6 (21)	3.3±0.1 (22)	0.41±0.02 (30)*	N.D.	47.3±3.5 (18)
Female K/O	20.2±0.2 (14)	96.1±0.3 (12)*	122.1±4.9 (14)	64.0±2.2 (11)	46.7±4.4 (11)	3.3±0.3 (11)	0.45±0.03 (14)**	16.0±2.8 (11)	38.3±5.5 (9)
Male WT	26.3±0.2 (24)	101±0.3 (24)	133.1±4.7 (23)	74.5±1.5 (13)	46.6±2.1 (13)	2.8±0.2 (13)	0.67±0.04 (19)	60.1±7.5 (17)	42.4±2.0 (12)
Male^−/+^	26.1±0.2 (49)	100.5±0.2 (48)	135.7±2.6 (49)	68.5±1.0 (31)*	45.9±1.4 (32)	2.8±0.1 (31)	0.65±0.03 (37)	N.D.	39.4±2.8 (29)
Male K/O	26.0±0.4 (27)	100.7±0.3 (26)	135.0±4.5 (27)	72.5±1.4 (19)	47.5±2.1 (19)	2.9±0.2 (20)	0.60±0.04 (24)	44.1±3.8 (22)*	38.8±2.9 (19)

Length: *F WT vs. F KO, p<0.01; Cholesterol: *M WT vs. M Het p<0.01; Insulin: *F WT vs. F Het, p<0.001; **F WT vs. F KO, p<0.05; Proinsulin: *M WT vs. KO, p<0.05.

At 16 weeks of age mice were fasted for 5 hours and then weighed. One hour later mice were anesthetized, their length was measured and blood isolated. Blood glucose and plasma cholesterol, triacylglycerol, glycerol, insulin and glucagon levels were determined as described in Materials and Methods. Results represent mean data ± S.E.M. obtained from the indicated number of animals in parentheses.

### Insulin Secretion from Male C57BL/6J *Slc30a8* KO Mouse Islets

**Figure 3:**
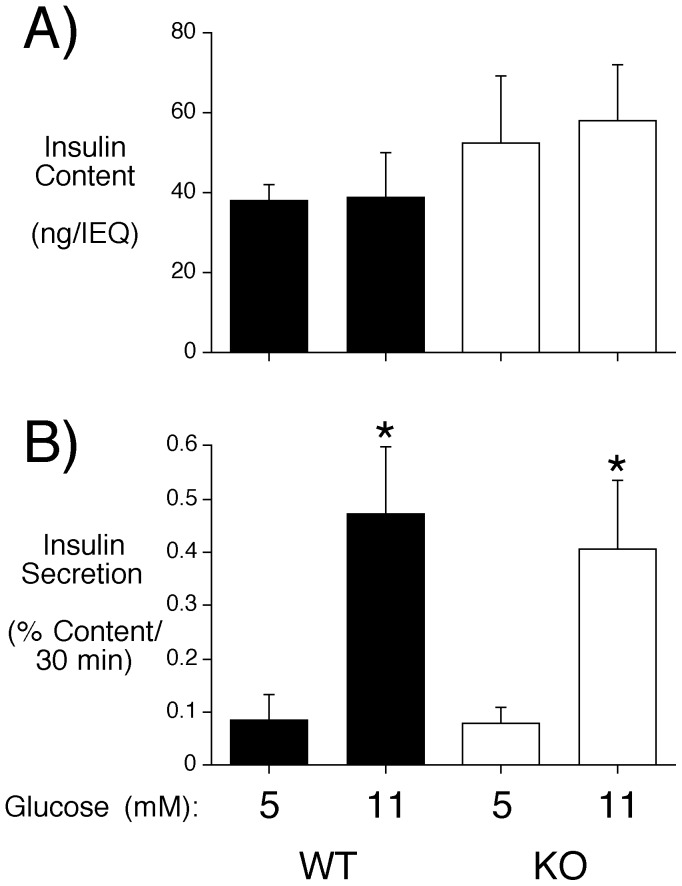
Analysis of insulin content and glucose-stimulated insulin secretion in male C57BL/6J *Slc30a8* KO mouse islets *in situ*. Islets were isolated from male C57BL/6J WT and *Slc30a8* KO mice and then insulin content (**Panel A**) and glucose-stimulated insulin secretion (**Panel B**) were assayed as described in Materials and Methods. Results show the mean data ± S.E.M. from 3 islet preparations isolated from ∼18 week old male mice. *p<0.05 *versus* 5 mM glucose.

Even though fasting plasma insulin and glucose were unaltered in male C57BL/6J *Slc30a8* KO mice (Table 1), we next analyzed GSIS in islets isolated from ∼18 week old male mice so as to allow a direct comparison with the equivalent data obtained with islets isolated from *Slc30a8* KO mice on a mixed genetic background. Following overnight culture in 5 mM glucose, islets were incubated in either 5 mM or 11 mM glucose for 30 min. No change in insulin content was observed in C57BL/6J *Slc30a8* KO mouse islets (Fig. 3A), consistent with data obtained with islets isolated from *Slc30a8* KO mice on a mixed genetic background [17]. However, in marked contrast to the reduced GSIS observed using islets isolated from *Slc30a8* KO mice on a mixed genetic background [17], islets from male C57BL/6J *Slc30a8* KO mice displayed no change in GSIS (Fig. 3B). These data are again consistent with the conclusion that the phenotype of male *Slc30a8* KO mice is dependent upon 129SvEv-specific modifier genes.

### Phenotypic Characterization of High Fat Fed Male C57BL/6J *Slc30a8* KO Mice

**Figure 4:**
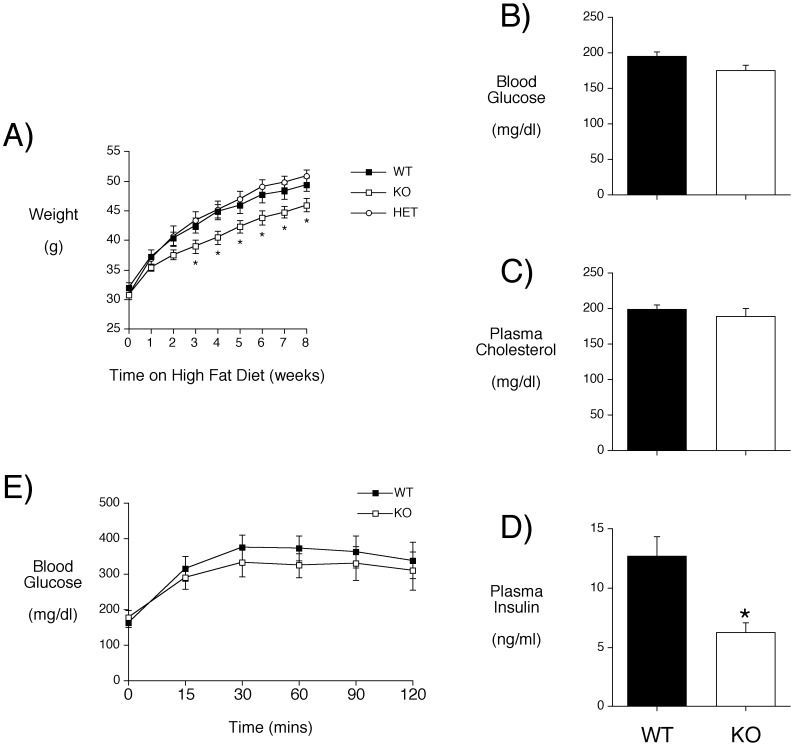
Phenotypic characterization of high fat fed male C57BL/6J *Slc30a8* KO mice. Male WT (n = 5; mean age 44.6 weeks), heterozygous (HET) (n = 14; mean age 39.6 weeks) and KO (n = 6; mean age 49.5 weeks) mice were fed a high fat diet with body weights measured weekly (**Panel A**). After 8 weeks mice fasted for 4 hours and then weighed. One hour later mice were anesthetized and blood was isolated. Blood glucose (**Panel B**) and plasma cholesterol (**Panel C**) and insulin (**Panel D**) levels were determined as described in Materials and Methods. One week later IPGTTs were performed on 6 hr fasted conscious C57BL/6J WT (closed symbols) and *Slc30a8* KO (open symbols) male mice as described in Materials and Methods (Panel **E**). Results represent mean data ± S.E.M. *p<0.05 *versus* WT.

We next investigated the effect of high-fat feeding, a standard nutritional challenge in the field of obesity and diabetes research that induces insulin resistance and is considered to model human disease [33]. We specifically studied older mice (∼40–50 weeks old) since susceptibility to type 2 diabetes increases with age [34]. High fat feeding was started at ∼40–50 weeks of age and continued for 8 weeks. Figure 4A shows that C57BL/6J *Slc30a8* KO mice were protected from diet-induced obesity, relative to WT and heterozygous mice. Fasting plasma triglyceride levels were only slightly higher in chow fed compared to high fat fed mice (WT: 46.6±2.1 vs 52.0±1.4; KO: 47.5±2.1 vs 55.3±6.6) whereas fasting blood glucose (Fig. 4B), plasma cholesterol (Fig. 4C) and plasma insulin (Fig. 4D) were all markedly elevated in both high fat fed WT and KO mice compared to chow fed animals (Table 1). These changes are consistent with the presence of insulin resistance though, because the ages of the chow fed and high fat fed animals differ, it is unclear to what degree these differences are due to age rather than diet. In high fat fed mice there was no difference in fasting blood glucose (Fig. 4B) or plasma cholesterol (Fig. 4C) between high fat fed WT and C57BL/6J *Slc30a8* KO mice whereas fasting plasma insulin was much higher in WT mice (Fig. 4D), consistent with the greater weight of WT mice (Fig. 4A). However, despite less adiposity and lower fasting insulin concentrations in the C57BL/6J *Slc30a8* KO mice, this did not result in a marked improvement in glucose tolerance (Fig. 4E).

### Phenotypic Characterization of Fasted Female C57BL/6J *Slc30a8* KO Mice

Although the phenotype of male *Slc30a8* KO mice observed on a mixed genetic background was not retained on the C57BL/6J background, female *Slc30a8* KO mice on the mixed genetic background also displayed reduced fasting insulin levels [17]. We therefore assessed the phenotype of the female C57BL/6J *Slc30a8* KO mice following a 6 hour fast at ∼16 weeks of age (Table 1). As with the males, female C57BL/6J *Slc30a8* KO mice displayed no marked differences in body weight or length or fasting plasma cholesterol, glycerol, triglycerides or glucagon. But, consistent with observations on the mixed genetic background, female C57BL/6J *Slc30a8* KO mice displayed a marked reduction (∼20%) in fasting plasma insulin levels with no change in fasting blood glucose levels (Table 1), though the magnitude of this reduction was somewhat greater in female *Slc30a8* KO mice on the mixed genetic background (∼30%) [17]. This result implies that, in contrast to male mice, the phenotype of female *Slc30a8* KO mice is less dependent upon the influence of 129SvEv-specific modifier genes.

### Phenotypic Characterization of Female C57BL/6J *Slc30a8* KO Mice Following a Glucose Challenge

**Figure 5:**
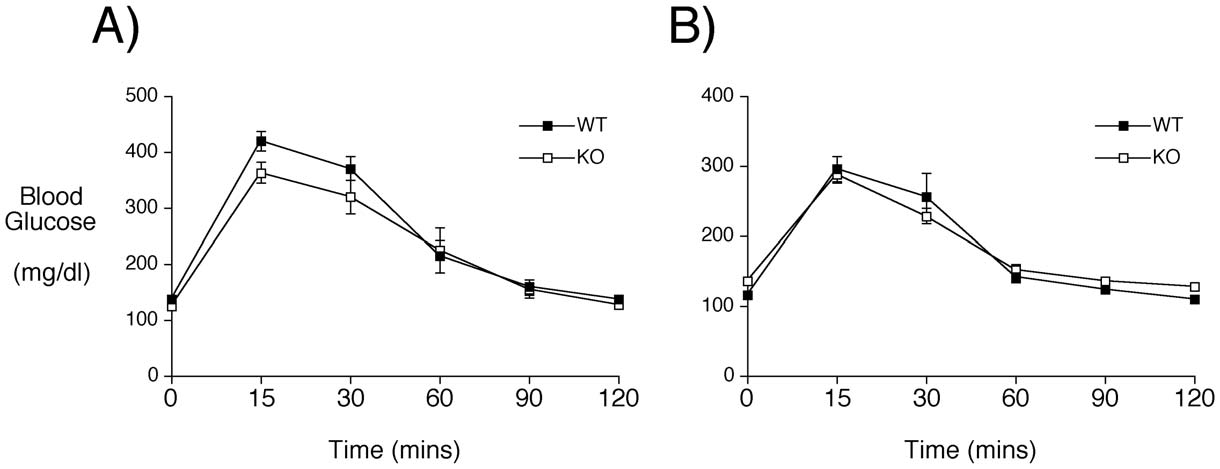
Analysis of glucose tolerance in female C57BL/6J *Slc30a8* KO mice *in vivo*. Intraperitoneal (Panel **A**) and oral (Panel **B**) glucose tolerance tests were performed on 6 hr fasted conscious C57BL/6J WT (closed symbols) and *Slc30a8* KO (open symbols) female mice as described in Materials and Methods. The IPGTT results show the mean glucose concentrations ± S.E.M. in WT (n = 6; mean age ∼20 weeks) and *Slc30a8* KO (n = 8; mean age ∼20 weeks) animals. The OGTT results show the mean glucose concentrations ± S.E.M. in WT (n = 9; mean age ∼22 weeks) and *Slc30a8* KO (n = 12; mean age ∼22 weeks) animals.

We next determined whether the reduction in fasting plasma insulin in female C57BL/6J *Slc30a8* KO mice resulted in impaired glucose tolerance. Both intraperitoneal (Fig. 5A) and oral (Fig. 5B) glucose tolerance tests indicated that glucose tolerance is normal in female C57BL/6J *Slc30a8* KO mice. A similar observation was previously made with male *Slc30a8* KO mice on a mixed genetic background in which a marked reduction in fasting plasma insulin was not associated with a change in glucose tolerance [17].

### Insulin Secretion from Female C57BL/6J *Slc30a8* Mouse Islets

**Figure 6:**
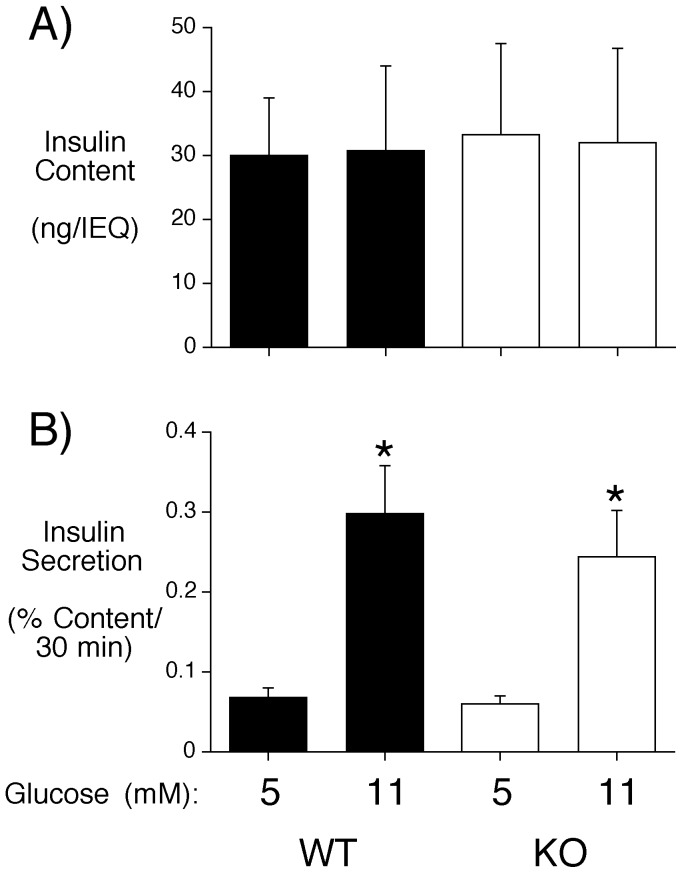
Analysis of insulin content and GSIS in female C57BL/6J *Slc30a8* KO mouse islets *in situ*. Islets were isolated from female WT and *Slc30a8* KO mice and then insulin content (**Panel A**) and GSIS (**Panel B**) were assayed as described in Materials and Methods. Results show the mean data ± S.E.M. from 6 islet preparations isolated from ∼18 week old female mice. *p<0.05 *versus* 5 mM glucose.

With male *Slc30a8* KO mice on a mixed genetic background although glucose tolerance was not altered the marked reduction in fasting plasma insulin was associated with impaired GSIS [17]. We therefore investigated whether the reduction in fasting plasma insulin in female *Slc30a8* KO mice was also associated with impaired GSIS. Islets were isolated from ∼18 week old female C57BL/6J WT and *Slc30a8* KO mice and insulin content and secretion were measured following static incubations in either 5 mM or 11 mM glucose. No differences in insulin content between WT and KO islets were observed (Fig. 6A). And surprisingly, despite the reduction in fasting insulin levels, GSIS in female C57BL/6J *Slc30a8* KO mouse islets did not differ from WT islets (Fig. 6B). Thus, in contrast to male *Slc30a8* KO mice on the mixed genetic background, reduced fasting insulin levels were not associated with reduced GSIS from isolated female C57BL/6J *Slc30a8* KO mouse islets.

### Analysis of the Mechanism by which Fasting Plasma Insulin is Reduced in Female C57BL/6J *Slc30a8* KO Mice with no Change in Glucose Tolerance

To address the issue as to how fasting plasma insulin levels could be altered with no change in fasting blood glucose, we considered three possibilities. First, it was possible that female C57BL/6J *Slc30a8* KO mice would also manifest a corresponding reduction in fasting plasma glucagon levels, such that the KO mice are simply living at a different metabolic equilibrium. However, no significant difference in fasting plasma glucagon levels was observed (Table 1). This is consistent with the female *Slc30a8* KO mice on a mixed genetic background in which a reduction in fasting insulin was not associated with a change in fasting glucagon [17].

Second, because it has been proposed that zinc is important for proinsulin to insulin conversion [6] and, in humans, variations in the *SLC30A8* gene are associated with impaired proinsulin conversion [14], it was conceivable that increased proinsulin levels may have compensated for the reduction in insulin secretion. Proinsulin can bind, albeit with low affinity relative to insulin, to the insulin receptor and thus activate insulin signaling pathways [35]. However, fasting proinsulin levels were unchanged in female *Slc30a8* KO mice following a 6 hour fast (Table 1), though the difference between WT and KO was close to significance (P<0.06). Interestingly, male C57BL/6J *Slc30a8* KO mice displayed a significant reduction in proinsulin levels following a 6 hour fast, though this was not associated with a reduction in fasting glucose or insulin levels (Table 1). However, even this effect appears subject to the influence of modifier genes since it is not observed in male *Slc30a8* KO mice on a mixed genetic background (Fig. S1).

**Figure 7:**
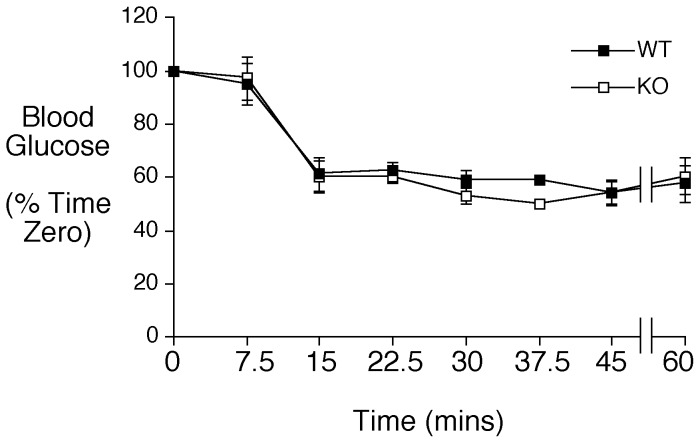
Analysis of insulin sensitivity in female C57BL/6J *Slc30a8* KO mice *in vivo*. Insulin tolerance tests were performed on 5 hr fasted conscious C57BL/6J WT (closed symbols) and *Slc30a8* KO (open symbols) female mice as described in Materials and Methods. Results show the mean glucose concentrations ± S.E.M. in WT (n = 8; mean age ∼18 weeks) and *Slc30a8* KO (n = 8; mean age ∼18 weeks) animals. In these groups of mice the initial glucose concentration at time zero was higher in KO mice (142.6±6.1 vs 120.8±4.9 mg/dl; p<0.02).

Finally, we considered the possibility that insulin sensitivity was enhanced in the female C57BL/6J *Slc30a8* KO mice to offset the reduction in fasting insulin secretion. Such a change could have arisen either indirectly, as an adaptive compensation to offset the reduction in fasting insulin secretion, or directly as a consequence of the absence of ZnT-8 in several other tissues where it is expressed at low levels [4]. Insulin tolerance tests, however, indicated that neither female (Fig. 7) nor male (Fig. S2) C57BL/6J *Slc30a8* KO mice display altered insulin sensitivity.

### Analysis of C57BL/6J *Slc30a8* KO Mouse Islets Using Electron Microscopy

Previous studies [18]–[20] have reported markedly altered islet secretory granule morphology in *Slc30a8* KO mice, which seems at odds with the mild metabolic phenotype. We therefore re-examined islet beta cell secretory granule morphology in the C57BL/6J *Slc30a8* KO mice. The results demonstrate that female C57BL/6J *Slc30a8* KO granules are not markedly different from those in control mice (Fig. S3). The size and shape of both the granules and the electron dense cores were similar to those from control islets (Fig. S3). The explanation for these apparently conflicting data is unclear. In the published studies Nicholson et al. [20] and Wijesekara et al. [18] both fixed isolated islets whereas Lemaire et al. [19] fixed intact pancreas by immersion. In contrast, our fixation method involved the more challenging fixation of the pancreas by vascular perfusion *in situ*. This method rapidly and uniformly delivers the fixative to the tissue using the animals’ own vasculature. However, we think it unlikely that the fixation method used would explain the differences between our results and published observations.

### Analysis of Pancreatic *SLC30* Developmental Expression

**Figure 8:**
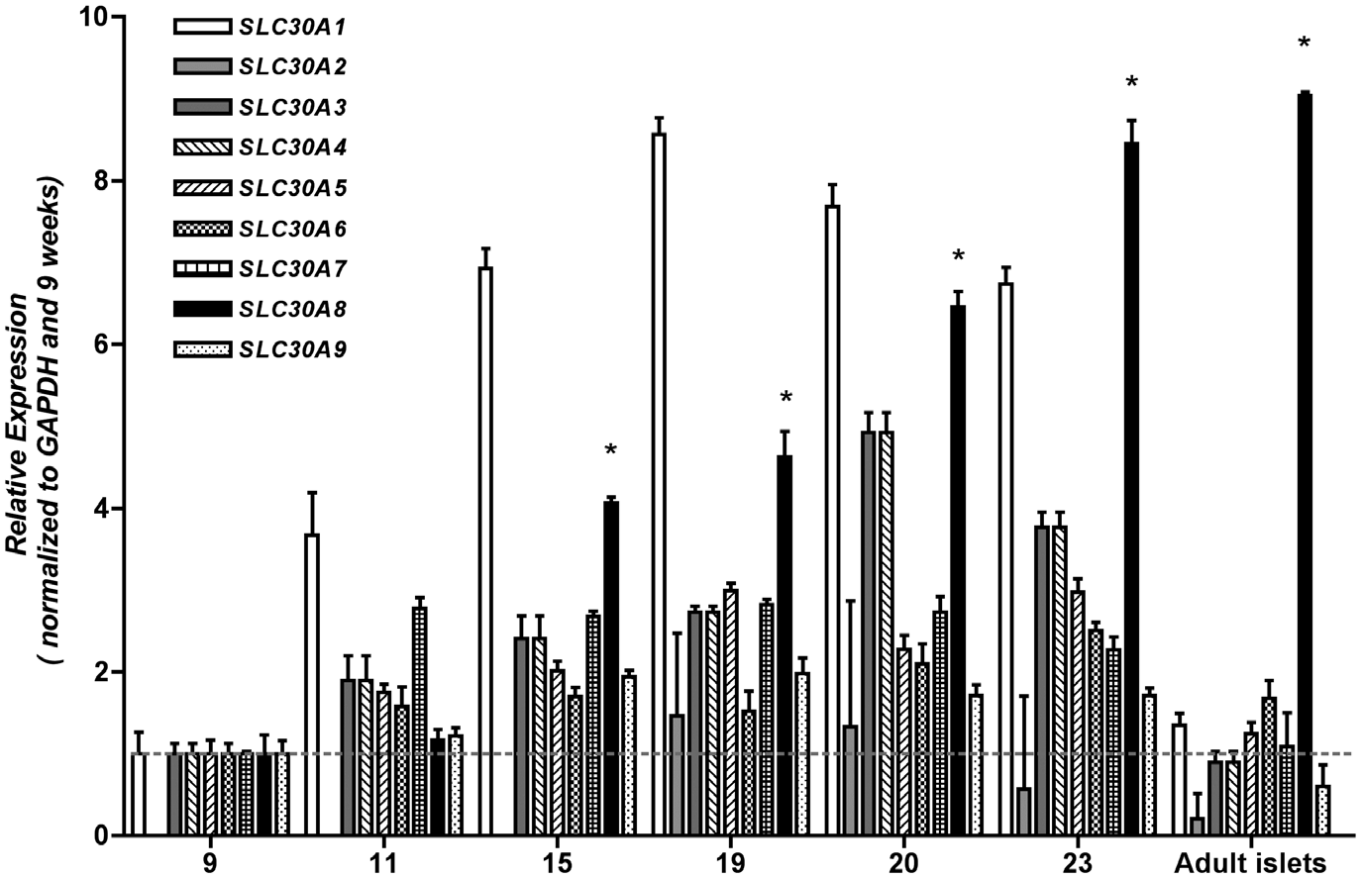
Normalized expression of selected *SLC30* genes by quantitative RT-PCR in 9–23 week old human fetal pancreas and adult human islets. Data in triplicate (mean ± S.E.M.) is normalized to endogenous *GAPDH* and quantified and expressed relative to 9 week old fetal samples. *SLC30A2* expression was detectable in the fetal pancreas from 19 weeks. *p<0.05 from 9 weeks for *SLC30A8* (black bars).

The association between *SLC30A8* and susceptibility to type 2 diabetes appears at odds with the very mild phenotype of *Slc30a8* KO mice. While there are multiple possible explanations for this apparent inconsistency we considered the possibility that ZnT-8 may play a more important role during islet development in humans than mice. Indeed, islet development appears significantly different between humans and mice with the human equivalent of a mouse endocrine secondary transition not evident, either in terms of morphology or in dramatic changes in endocrine-specific transcriptional regulators [31]. We have previously shown that a marked increase in islet *Slc30a8* gene expression is observed between e15.5 and e17.5 in mice whereas *insulin* expression is already clearly evident at e15.5 [27]. We have also previously shown that in humans *insulin*, *glucagon*, *somatostatin*, *ghrelin* and *pancreatic polypeptide* transcripts are already present at 9–10 weeks and only increase ∼50% further by 23 weeks, commensurate with the expansion of endocrine cell volume [31]. Figure 8 shows, using the identical human RNA samples as used for this previous study, that *SLC30A8* expression increases markedly between 9 weeks and 23 weeks, indicating that in humans as in mice, high *insulin* expression precedes that of *SLC30A8*. Figure 8 also shows that *SLC30A8* is the major *SLC30* isoform in human islets, as it is in mice [20]. These observations do not support the concept of a dramatic difference between mice and humans with respect to a role for ZnT-8 during development.

## Discussion

Our data suggest that the consequences of *Slc30a8* gene deletion in mice are both gender- and genetic background-specific. A decrease in fasting insulin was observed in female *Slc30a8* KO mice on both the pure C57BL/6J and mixed genetic backgrounds whereas this difference was only observed in male *Slc30a8* KO mice on the mixed genetic background (Table 1) [17]. In male *Slc30a8* KO mice on a mixed genetic background this decrease in fasting insulin was associated with reduced GSIS from isolated islets [17] whereas in female *Slc30a8* KO mice on the C57BL/6J genetic background it was not (Fig. 6). While this may indicate that a defect that exists in female *Slc30a8* KO islets *in vivo* is not maintained in isolated islets, this apparent discrepancy more likely simply reflects the fact that the decrease in fasting insulin in male *Slc30a8* KO mice on a mixed genetic background is much greater than seen in fasting C57BL/6J female *Slc30a8* KO mice (50% vs 20%; Ref. [17] and Table 1). Therefore many more isolated islet preparations would have to be studied to uncover the expected small change in GSIS, which is not feasible given the inherent variability between isolated islet preparations. Most strikingly, no change in glucose tolerance was observed in male or female *Slc30a8* KO mice on either a mixed [17] or pure (Figs. 2 & 5) genetic background. Overall these results suggest a minor role for *Slc30a8* and islet zinc in the regulation of glucose metabolism. The small alteration in proinsulin secretion in male *Slc30a8* KO mice (Table 1) does perhaps suggest a role for zinc in insulin processing, though even this effect appears to be influenced by genetic background (Fig. S1).

The conclusion from this study that ZnT-8 plays a minor role in the regulation of glucose metabolism is consistent with our previous conclusion [17] and that of Lemaire et al. [19]. In contrast, Nicholson et al. [20] observed an impairment in glucose tolerance and insulin secretion in *Slc30a8* KO mice during IPGTTs. All these studies were performed in mice with a global *Slc30a8* deletion on a mixed genetic background. In more recent studies in which the *Slc30a8* gene was deleted specifically in beta cells, the same group observed a more mild impairment in glucose tolerance during OGTTs [18]. While this mild impairment in glucose tolerance was not associated with an impairment of insulin secretion *in vivo*, an impairment in first phase insulin secretion was observed in isolated islets *in vitro* [18]. In humans the rs13266634 polymorphism in the *SLC30A8* gene has been linked to not only type 2 diabetes but also impaired glucose tolerance [36], impaired proinsulin to insulin conversion [14] and reduced first phase insulin secretion [15]. It is therefore apparent that a change in ZnT-8 function in humans leads to impaired beta cell function, making it easy to envisage how a functionally impaired beta cell would be more prone to the action of other environmental factors that promote beta cell failure and lead to type 2 diabetes [37]. In contrast, overall, the studies of ZnT-8 function in mice fail to clearly establish why this protein would be associated with altered susceptibility to the development of type 2 diabetes. There is clear evidence for altered zinc levels [17]–[20], GSIS [17], [18] and proinsulin conversion (Fig. S1; Ref. [18]) in these mouse models but the effects are influenced by gender and modifier genes and/or environmental differences such that there is no consistent change in glucose tolerance. These observations initially suggested an inherent difference between humans and mice with respect to the importance of ZnT-8 for islet function or the ability to compensate for changes in ZnT-8 expression. However it has recently been shown that, while the rs13266634 *SLC30A8* variant is associated with type 2 diabetes risk in Asians and Europeans, no association is observed in Africans [36]. This suggests that modifier genes and/or environmental differences also influence the effect of *SLC30A8* variations in humans.

In contrast to many mouse models of insulin resistance, high fat feeding did not unmask a diabetic phenotype in male C57BL/6J *Slc30a8* KO mice, in fact the KO mice were protected from diet-induced obesity (Fig. 4A). Two studies have previously investigated the effect of a high fat diet on the phenotype of *Slc30a8* KO mice on a mixed genetic background [19], [20]. In contrast to our findings, one study found that prolonged high fat feeding caused a greater weight gain in *Slc30a8* KO mice, though the WT mice in this study gained less than 10 g despite 13 weeks of high fat feeding [20], and the other study reported no statistically significant differences [19]. Furthermore, although we noted a significant decrease in fasting plasma insulin levels in C57BL/6J *Slc30a8* KO mice (Fig. 4D), Nicolson *et al*. [20] observed that *Slc30a8* KO mice displayed increased fasting plasma insulin levels. The explanation for these differences is unclear though variables include genetic background and the age of the mice studied, with both Nicolson *et al*. [20] and Lemaire et al. [19] examining the effect of high fat feeding in young mice (high fat diet initiated at 6 weeks of age) in contrast to the old mice (high fat diet initiated at ∼40 weeks of age) studied here. Neither Nicolson *et al*. [20] nor Lemaire et al. [19] detected a statistically significant change in glucose tolerance in high fat fed *Slc30a8* KO mice, consistent with our observations (Fig. 4E). However Lemaire et al. [19] reported that longer exposure to a high fat diet did result in the appearance of overt diabetes in two out of four *Slc30a8* KO mice, while under the same conditions all eight of the control mice studied remained non-diabetic. More recently, Hardy et al. [38] compared the effects of high-fat high-calorie diet feeding on mice with *Slc30a8* deleted globally or selectively in beta cells. In this study mice with a global deletion of *Slc30a8* became obese, hyperglycemic, hyperinsulinemic, insulin resistant, and glucose intolerant compared with littermate controls [38], similar to the results of Nicholson et al. [20]. In contrast, mice with *Slc30a8* deleted selectively in beta cells had impaired glucose tolerance, though similar body weights, compared with littermate controls [38]. These data suggest that ZnT-8 contributes to the risk of developing type 2 diabetes through both beta cell-dependent and independent effects, though the authors note that some of the observed differences might be due to differences in the genetic backgrounds of the mice studied [38].

The differences between these studies have been attributed to variations in the age and genetic background of the mice examined [21]. However, there can also be significant variations in glucose metabolism between genetically identical lines housed within different mouse facilities such that it is possible that some of the discrepancies in the phenotype of *Slc30a8* KO mice reported by different groups are due to environmental differences [24]. However, the observed differences between our present studies and our previous studies [17] are more likely to be to due to differences in genetic background given that the animals were housed in the same facility.

The physiological basis for observed reduction in fasting insulin levels in female mice on the pure C57BL/6J background (Table 1), and both male and female mice on the mixed genetic background [17], without a concomitant change in fasting glucose levels remains unknown. This phenomenon does not appear to be explained by altered insulin sensitivity (Fig. 7) or a major change in proinsulin secretion (Table 1). The results do provide weak evidence for an offsetting change in glucagon secretion. Thus, though no statistically significant changes in fasting glucagon levels were observed in male or female C57BL/6J *Slc30a8* KO mice, female C57BL/6J *Slc30a8* KO mice do display a trend toward reduced glucagon secretion (Table 1). Furthermore, this trend is also observed in male mice on a mixed genetic background and reaches significance in the comparison of KO and heterozygous mice, presumably due to the larger sample size [17]. This might suggest that our inability to detect a difference in fasting glucagon levels in the aforementioned groups may be due to a lack of power. The hypothesis that glucagon secretion is impaired in *Slc30a8* KO mice would be consistent with the demonstration that ZnT-8 is expressed in alpha cells [17]. In addition, it has been previously shown that the zinc released from the insulin hexamers following insulin release by the beta cell can affect glucagon secretion by the alpha cell, though the mechanism by which it does this is disputed [3], [39], [40]. This suggests that deletion of *Slc30a8* could affect glucagon secretion from alpha cells by both direct and indirect mechanisms. However, in fasting female *Slc30a8* KO mice on a mixed genetic background there is no evidence for a trend toward reduced glucagon secretion despite the clear reduction in fasting insulin [17], which implies that altered glucagon secretion cannot explain the normal fasting glucose levels in the presence of reduced fasting insulin levels.

Overall, these observations suggest that the complete absence of ZnT-8 is not enough to lead to impaired glucose tolerance in mice. It is conceivable, however, that additional disturbances are required to unmask a more significant phenotype in *Slc30a8* KO mice, such the presence of mutations in other type 2 diabetes-associated genes or zinc-deficient diets. Future studies will address these possibilities. Examining the effect of *Slc30a8* deletion in adult mice rather than during development might also be informative. The results also suggest that either very low levels of zinc are sufficient for proper insulin secretion or that zinc is not as important for insulin secretion as previously hypothesized [41]. Indeed, guinea-pig insulin lacks a histidine residue in the B10 position of the molecule, which normally binds zinc in hexamer formation, and the zinc content of guinea pig islets is very low compared to mice and rats [42].

## Supporting Information

Figure S1 **Analysis of plasma proinsulin in mixed genetic background** ***Slc30a8*** **KO mice** ***in vivo*** **.** At 16 weeks of age mice were fasted for 5 hours and then weighed. One hour later mice were anesthetized, their length was measured and blood isolated. Plasma proinsulin levels were determined as described in Materials and Methods. Results represent mean data ± S.E.M. obtained from the indicated number of animals in parentheses.
(TIFF)

Figure S2 **Analysis of insulin sensitivity in male C57BL/6J** ***Slc30a8*** **KO mice** ***in vivo*** **.** Insulin tolerance tests were performed on 5 hr fasted conscious C57BL/6J WT (closed symbols) and *Slc30a8* KO (open symbols) male mice as described in Materials and Methods. Results show the mean glucose concentrations ± S.E.M. in WT (n = 11; mean age ∼18 weeks) and *Slc30a8* KO (n = 6; mean age ∼18 weeks) animals. In these groups of mice the initial glucose concentration at time zero was no different between WT (153.0±4.8 mg/dl) and KO (165.0±2.3 mg/dl) mice.
(TIFF)

Figure S3 **Analysis of insulin secretory granule structure in C57BL/6J** ***Slc30a8*** **KO mice.** Islets were fixed *in situ* using pancreas perfusion and electron microscopy was then performed on pancreas sections as described in Materials and Methods.
(TIFF)
